# Ultrasound-controlled MXene-based Schottky heterojunction improves anti-infection and osteogenesis properties

**DOI:** 10.7150/thno.81511

**Published:** 2023-03-13

**Authors:** Hongchuan Wang, Na Mu, Yaqi He, Xiaoguang Zhang, Jie Lei, Cao Yang, Liang Ma, Yong Gao

**Affiliations:** 1 Department of Orthopaedics, Union Hospital, Tongji Medical College, Huazhong University of Science and Technology, Wuhan 430022, China.; 2 College of Agronomy, Xinjiang Agriculture University, Urumqi, Xinjiang, China.

**Keywords:** Sonodynamic therapy, Antibacterial therapy, Schottky Heterojunction, Bone regeneration, Reactive oxygen species

## Abstract

**Background:** The current clinical treatment of osteomyelitis is limited by the emergence of drug-resistant bacteria, which often leads to the failure of traditional antibiotic treatment and large bone defects. Sonodynamic therapy (SDT) is a new strategy that is widely used to overcome the problem of bacterial resistance to antibiotic therapy as well as poor tissue penetration using near-infrared light in photodynamic therapy (PDT). Therefore, it is necessary to develop a new sonosensitizer that can kill bacteria and promote bone repair.

**Methods:** Herein, we developed a sonosensitizer, porphyrin metal-organic framework (HNTM), with a Schottky junction modified by Ti_3_C_2_ nanosheets (HN-Ti_3_C_2_) for highly efficient sonodynamic therapy of osteomyelitis and bone regeneration.

**Results:** Ti_3_C_2_ greatly improves the acoustic catalytic performance by rapidly transferring the charge carriers generated by HNTM under ultrasound (US) irradiation, which killing drug-resistant bacteria through the generation of large amounts of reactive oxygen species (ROS). And HN-Ti_3_C_2_ shows excellent 99.75% antibacterial effectiveness against MRSA. In addition, HN-Ti_3_C_2_ generates a sonocurrent under low-intensity US to promote the repair of bone defects for a long time period. Mechanistic research using CCK-8 and RNA-seq showed that HN-Ti_3_C_2_ nanocomposites can promote the proliferation of stem cells by regulating the cell cycle, DNA replication, and apoptosis. In addition, after low-intensity US irradiation, HN-Ti_3_C_2_ promotes osteogenic differentiation via some key signaling pathways, including the calcium, Wnt, and TGF-beta signaling pathways, according to the Kyoto Encyclopedia of Genes and Genomes (KEGG). In a MRSA-infected rat tibial osteomyelitis model, HN-Ti_3_C_2_ successfully eliminated the infection and significantly improved bone regeneration under US irradiation.

**Conclusion:** This study indicates that engineered HN-Ti_3_C_2_ is a distinctive nanocomposite for successful osteomyelitis treatment.

## Introduction

Infectious bacterial diseases remain a major public health challenge. Owing to the high variability of bacteria, large doses of antibiotics can cause the emergence of drug-resistant bacteria 1. Hydrogels and other carriers have been used to load and release antibiotics *in situ* to reduce antibiotic dosage and increase the concentration of antibiotics at the infection site. However, the low antibacterial efficiency of this strategy in the face of superbugs cannot meet the therapeutic needs 2, 3. Catalytically active materials excited by external stimuli have been extensively investigated in recent years 4, 5. For example, photodynamic therapy (PDT) has been widely used to antitumor and antibacterial therapy 6-8.

Osteomyelitis, a serious infective orthopedic disease, caused by microbial infection, often secondary to trauma or orthopedic surgery, and leads to large bone destruction 9-11. At present, the clinical treatment of osteomyelitis mainly includes use of high-dose antibiotics for a long time and repeated debridement 12. However, as mentioned above, large doses of antibiotics can cause the emergence of bacterial resistance, and long-term antibiotic treatment and invasive debridement can be detrimental to patient health 12-14. Therefore, many other treatments have been reported to replace antibiotic therapy for osteomyelitis, such as photodynamic, photothermal, and microwave (MV) therapy 15, 16. However, the weak tissue penetration of near infrared (NIR) radiation greatly reduces its therapeutic efficacy for deep-tissue infectious diseases such as osteomyelitis 8, 17. The emergence of sonodynamic therapy (SDT) provides an effective strategy for the infectious treatment in deep-tissue 18. Ultrasound (US) with excellent tissue penetration can kill pathogenic bacteria by irradiating sonosensitizers to produce reactive oxygen species (ROS) 19, 20. Many inorganic sonosensitizers that have been developed, such as TiO_2_, silicon NPs, ZnO NPs, and Ag_2_S quantum dots (QDs). However, these inorganic nanomaterials have poor biocompatibility and cannot biodegrade, limiting their clinical applications 21-25. In contrast, many organic sonosensitizers, such as porphyrins and their derivatives, 5-aminolevulinic acid, and phthalocyanines, have high sonocatalytic efficiency and good biocompatibility 26-28. However, the main disadvantages of these organic sonosensitizers are their poor stability and rapid degradation *in vivo* 29.

Metal-organic frameworks (MOFs) are very important compounds in which organic ligands link metal ions or metal clusters to form 3D coordination networks with potential holes 30. Owing to their highly adjustable composition, high porosity, and large surface area, MOFs are well-suited for the development of highly efficient catalysts 31, 32. In a previous study, we developed a MOF (HNTM) with the SDT effect by introducing porphyrin as an organic ligand. This strategy enhanced the stability of the organic sonosensitizer porphyrin *in vivo* 33. To avoid tissue damage caused by high-intensity US, it is necessary to further modify the sonosensitizer to improve its sonocatalytic efficiency under low-power US conditions. The transfer efficiency of the excited electrons is an important factor for the catalytic efficiency of sonosensitizers. The catalytic performance of photosensitizers and sonosensitizers can be significantly improved by introducing noble metals as co-catalysts to accelerate electron transfer 10, 34, 35. However, this not only increases the preparation cost but may also cause tissue toxicity due to the non-degradation of precious metals *in vivo* 36. Therefore, it is of great significance to find a cheap cocatalyst with high activity and good degradability for SDT *in vivo*.

MXenes are two-dimensional nanomaterial that includes transition metal carbides, nitrides, and carbonitrides 37. MXene nanosheets (NSs) can be easily obtained by selectively etching the A layer from MAX phage precursor 38. MXene possesses many hydrophilic functional groups (-O and-OH) on its surface 39, which makes it easy to establish strong connections with various semiconductors, and it has excellent metal conductivity to ensure efficient carrier transfer 40-42. These characteristics demonstrate their potential as cocatalysts 43, 44. Ti_3_C_2_ is the earliest synthesized MXene material and has been explored as a highly effective catalyst to improve the catalytic activity of photosensitizer 42, 45, 46. At the same time, previous studies have shown that Ti_3_C_2_ can be used to promote bone regeneration after bone tumor resection 47.

**Scheme 1:**
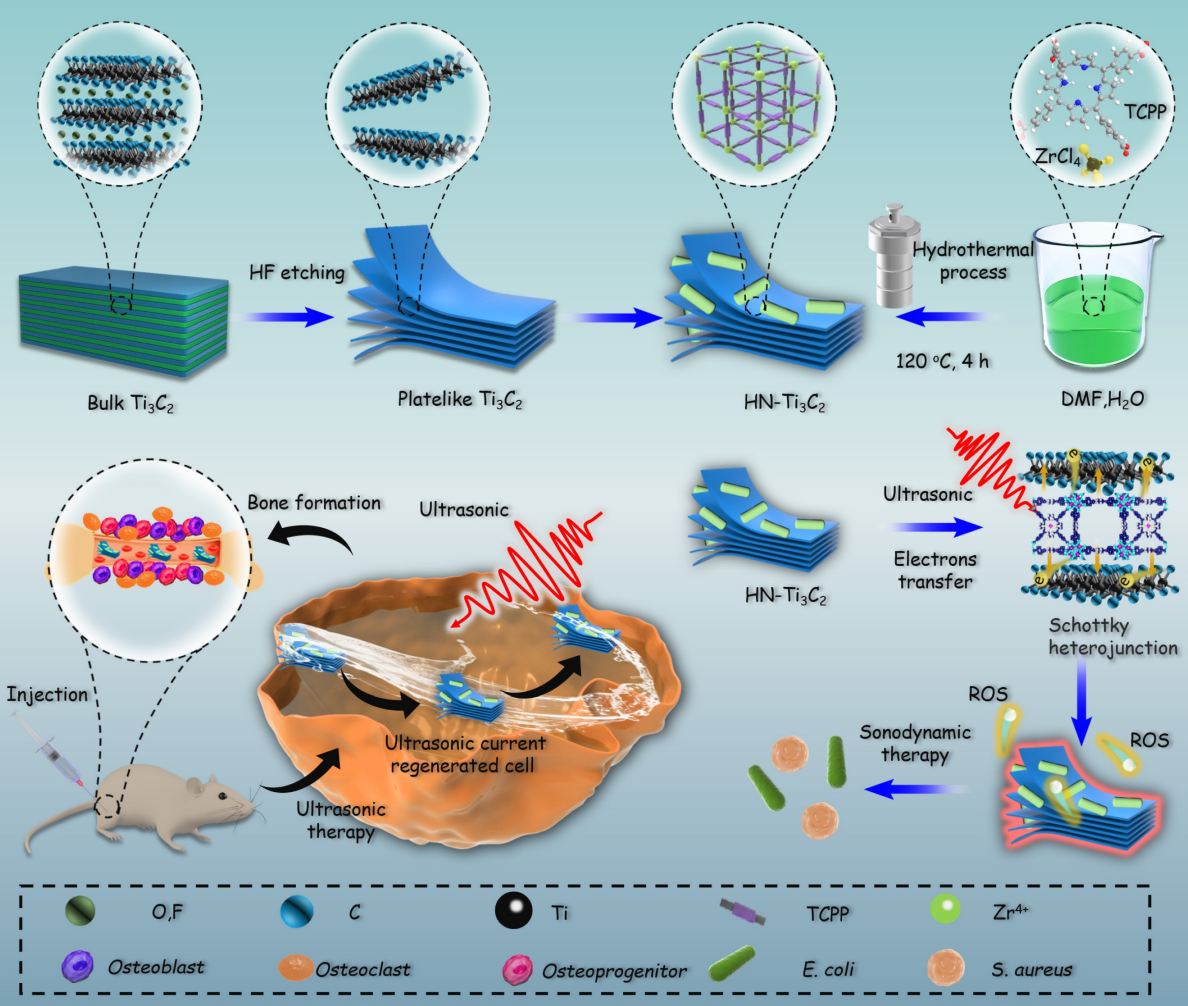
Mechanism of sonodynamic treatment of osteomyelitis.

Inspired by the above considerations, we synthesized HNTM/Ti_3_C_2_ hybrid nanomaterials (HN-Ti_3_C_2_) with Schottky heterojunctions for the treatment of osteomyelitis by SDT and promotion of bone regeneration. As shown in **Scheme 1**, HNTM generates abundant electron-hole pairs upon excitation by US, and Ti_3_C_2_ assists in the rapid transfer of electrons to generate large amounts of ROS to eliminate methicillin-resistant *Staphylococcus aureus* (MRSA) infection. According to the theory of acoustoelectric effect, when ultrasonic waves propagate in metals or semiconductors, mechanical energy interacts with electrons/carriers in the medium to generate electron motion and current 48. In this study, we found that HN-Ti_3_C_2_ can generate sonocurrent under low-intensity US to promote the repair of bone defects for a long time period. The gene set enrichment analysis (GSEA) of RNA-seq showed that HN-Ti_3_C_2_ may promote the proliferation of human bone marrow mesenchymal stem cells (hBMSCs) by regulating the cell cycle, DNA replication, and apoptosis. Kyoto Encyclopedia of Genes and Genomes (KEGG) analysis indicated that HN-Ti_3_C_2_ promotes osteogenic differentiation by some key signaling pathways, including the calcium, Wnt, and TGF-beta signaling pathways. In an MRSA-infected rat tibial osteomyelitis model, HN-Ti_3_C_2_ successfully eliminated the infection and significantly improved bone regeneration under US irradiation. In conclusion, we have provided a nano-hybrid material with anti-infection and bone-repair-promoting effects, which has achieved excellent efficacy in the treatment of osteomyelitis.

## Results and discussion

### Characterization of HNTM and HN-Ti_3_C_2_

**Figure 1:**
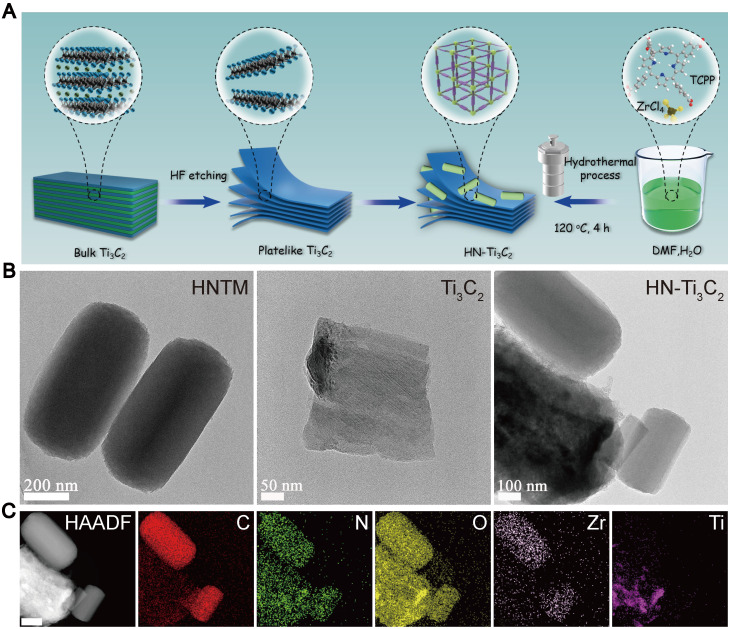
**Fabrication process and morphology characterization.** (A) Schematic illustration of the preparation procedure of Ti_3_C_2_, HNTM and HN-Ti_3_C_2_. (B) TEM images of HNTM, Ti_3_C_2_ and HN-Ti_3_C_2_. (C) Energy-dispersive X-ray spectroscopy elemental mapping of HN-Ti_3_C_2_. Scar bar: 200 nm.

The preparation processes for Ti_3_C_2_ NSs, HNTM, and HN-Ti_3_C_2_ are shown in **Figure 1A**. TEM was used to observe morphological characteristics. As shown in **Figure 1B**, HNTM exhibited a rod-like structure with a maximum diameter of 700 nm, whereas the 2D Ti_3_C_2_ NSs exhibited a lamellar structure. The observation of HN-Ti_3_C_2_ showed that HNTM and Ti_3_C_2_ NSs tightly adhered to each other, which illustrated the successful preparation of HN-Ti_3_C_2_. The tight combination of HNTM and Ti_3_C_2_ NSs was further characterized by the elemental mapping of C, N, O, Zr, and Ti **(Figure 1C)**. Then, HN-Ti_3_C_2_ was dispersed in PBS solution and soaked for 7 days to observe the morphology again by TEM. As shown in **Figure S1A**, HN-Ti_3_C_2_ can maintain its original morphology, which indicates its stability in PBS solution.

**Figure 2:**
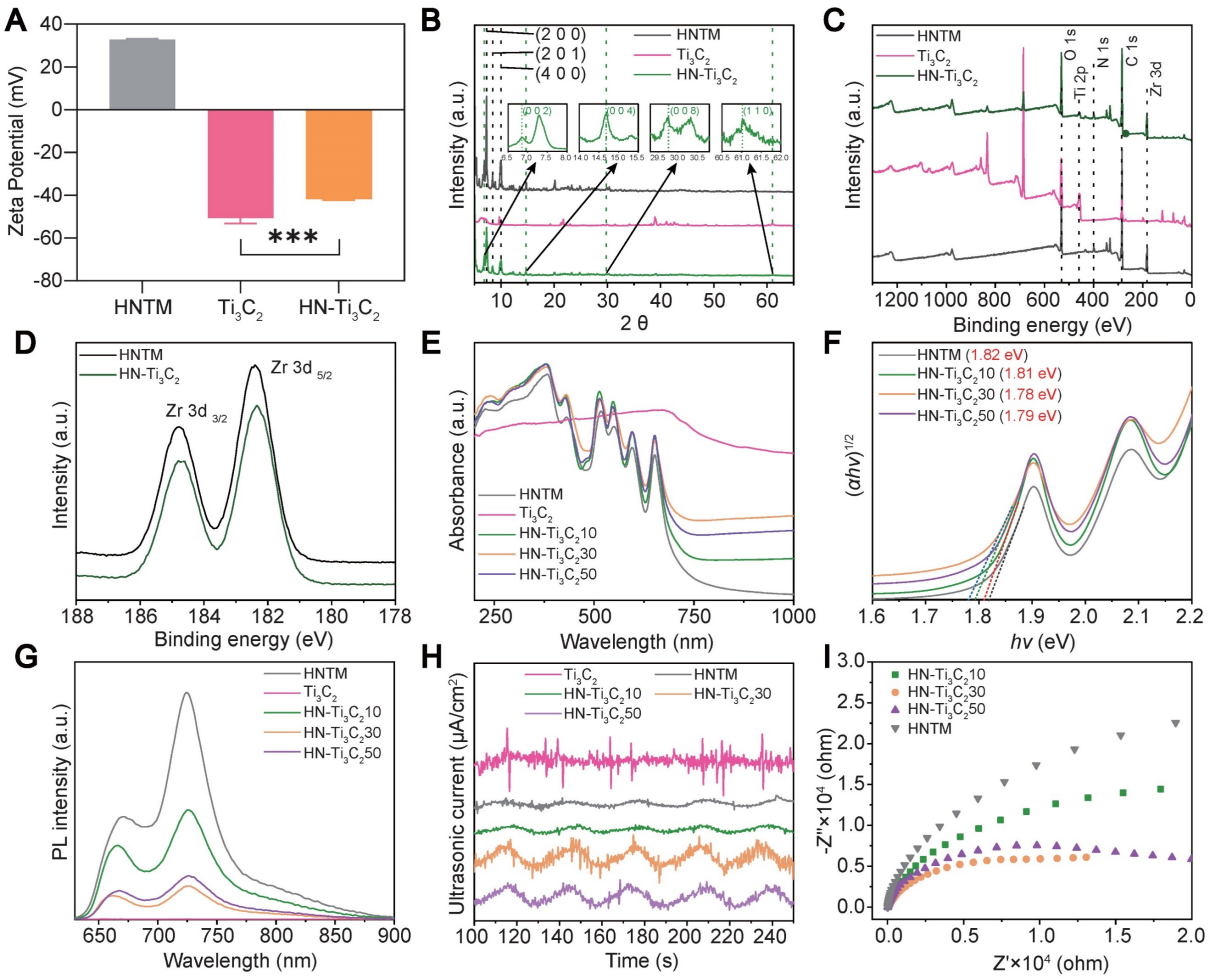
**Zeta potential, structural characterization and sonocatalytic detection.** (A) Zeta potential of HNTM, Ti_3_C_2_ and HN-Ti_3_C_2_. (B) XRD patterns of HNTM, Ti_3_C_2_ and HN-Ti_3_C_2_. (C) XPS of HNTM, Ti_3_C_2_ and HN-Ti_3_C_2_. (D) Zr 3d spectra of HNTM, and HN-Ti_3_C_2_. (E) UV-vis adsorption spectrum of HNTM, Ti_3_C_2_ and HN-Ti_3_C_2_ with different mass ratios. (F) Band gap of HNTM and HN-Ti_3_C_2_ with different mass ratios. (G) Photoluminescence spectra of HNTM, Ti_3_C_2_ and HN-Ti_3_C_2_ with different mass ratios. (H) Sonocurrent test of HNTM, Ti_3_C_2_ and HN-Ti_3_C_2_ with different mass ratios. (I) Electrochemical impedance measurement of HNTM and HN-Ti_3_C_2_ with different mass ratios. ***P < 0.001.

As shown in **Figure 2A**, the mean zeta potentials of HNTM and Ti_3_C_2_ were 32.8 mV and -50.6 mV, respectively. The zeta potential of HN-Ti_3_C_2_ was -41.8 mV after HNTM and Ti_3_C_2_ were combined, indicating that they were bound together by electrostatic interaction. Dynamic light scattering (DLS) measurements showed that the average hydrodynamic particle size of HNTM was about 600 nm (**Figure S1B**). Next, we investigated the crystal structure of the materials using XRD and found that HNTM had the same diffraction peaks as PCN-222 49. When HNTM was combined with Ti_3_C_2_, the diffraction peaks were significantly reduced. In HN-Ti_3_C_2_, diffraction peaks around 6.9°, 14.7°, 29.8°, and 61.0° can be assigned to (002), (004), (008), and (110) facets of Ti_3_C_2_ NSs** (Figure 2B** and **Figure S1C)** 50, 51. Main elements were detected by X-ray photoelectron spectroscopy (XPS) spectra in both HNTM and HN-Ti_3_C_2_, and in particular, the element Ti was detected in HN-Ti_3_C_2_ **(Figure 2C)**. In addition, two main peaks were observed in the Zr 3d spectra of both HNTM and HN-Ti_3_C_2_. Compared with HNTM, the two main peaks of HN-Ti_3_C_2_ shifted to the right, indicating that the Ti_3_C_2_ NSs altered the electron density of Zr atoms **(Figure 2D)**. We measured their UV-vis spectra, which showed that Ti_3_C_2_ NSs had a broad absorption spectrum from 200 to 1000 nm; however, the absorption spectrum of HNTM was significantly reduced after 650 nm. When HNTM was combined with Ti_3_C_2_ NSs at different mass ratios, the absorption spectra increased to different degrees, among which that of HN-Ti_3_C_2_30 increased the most significantly **(Figure 2E)**. As shown in **Figure 2F**, according to the relationship between Kubelka-Munk function and band gap energy, the band gaps of HNTM, HN-Ti_3_C_2_10, HN-Ti_3_C_2_30, and HN-Ti_3_C_2_50 were calculated to be 1.82, 1.81, 1.78, and 1.79 eV, respectively. These results indicate that HN-Ti_3_C_2_30 had the lowest band gap, and it was lower than most sonosensitizers in other studies, which resulted in easier electron generation under US irradiation 23, 52.

To compare the sonocatalytic activities of HNTM and HN-Ti_3_C_2_ with different mass ratios, we used photoluminescence (PL) spectroscopy to explore their electron-hole pair recombination rates. As shown in **Figure 2G**, the PL intensity of each HN-Ti_3_C_2_ group was significantly reduced compared to that of HNTM, indicating that combining with Ti_3_C_2_ NSs can inhibit electron-hole pair recombination in HNTM. Among them, HN-Ti_3_C_2_30 had the lowest PL intensity, indicating that HN-Ti_3_C_2_30 had the lowest electron-hole pair recombination efficiency and the highest sonocatalytic activity. To explore the sonocatalytic activity, the sonocurrent response and electrochemical impedance spectra of the samples were recorded using an electrochemical workstation. By repeating US irradiation (1.5 W/cm^2^, continuous, 1 MHz), we can see that an enhanced sonocurrent is generated after the combination with Ti_3_C_2_ NSs, and HN-Ti_3_C_2_30 has the strongest photocurrent. This result indicates that HN-Ti_3_C_2_30 generates more electrons and is transferred to Ti_3_C_2_ NSs under US irradiation **(Figure 2H)**. Similarly, the electrochemical impedance spectrum shows the smallest semicircular arc for HN-Ti_3_C_2_30, indicating the smallest electron transfer resistance, which is consistent with the current result **(Figure 2I)**.

**Figure 3:**
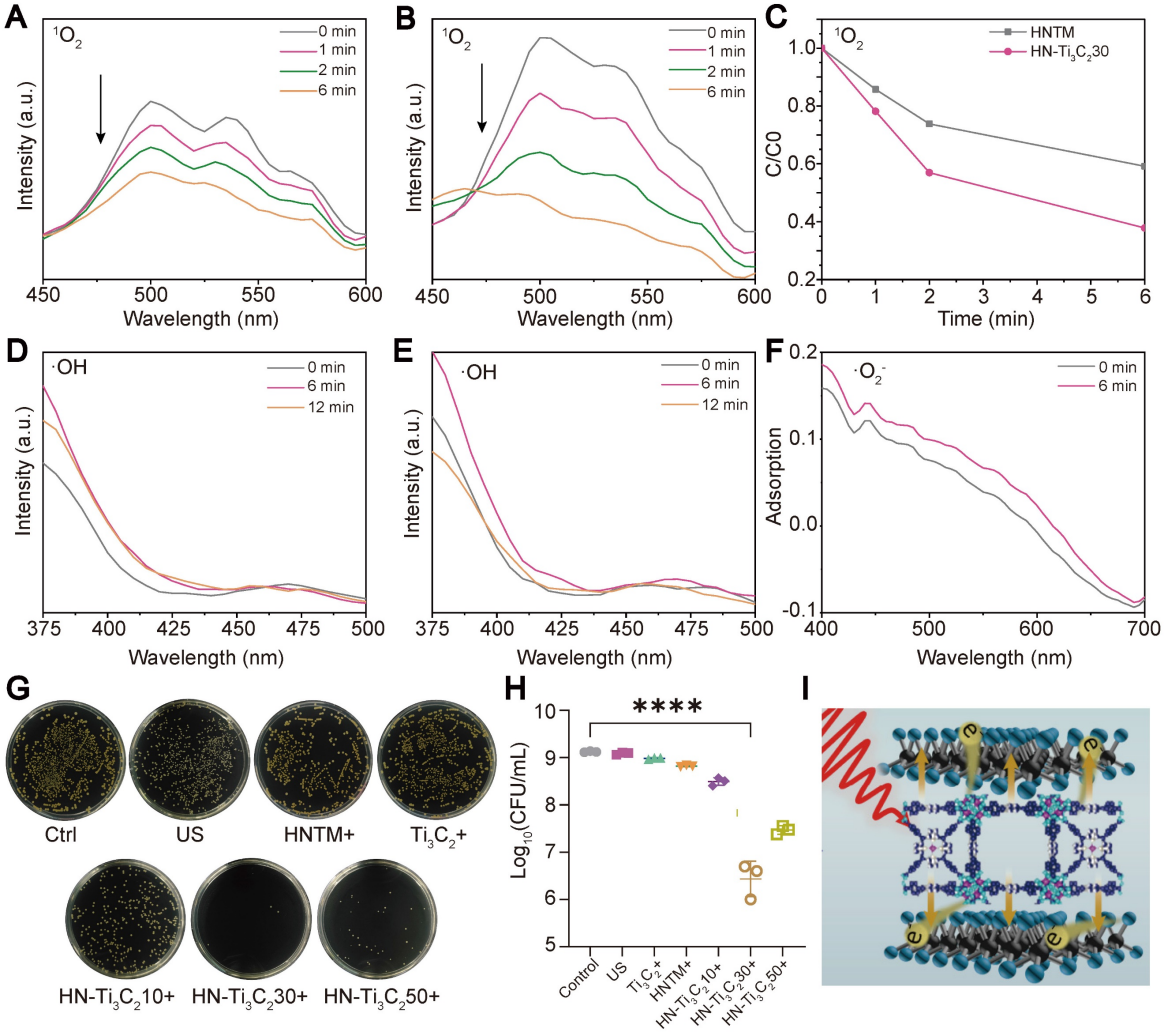
** ROS generation ability and *in vitro* antibacterial performance.** The ^1^O_2_ generation of (A) HNTM and (B) HN-Ti_3_C_2_30 under US detecting by the decrease of DMA fluorescence intensity. (C) Comparison curves of ^1^O_2_ generation capacity of HNTM and HN-Ti_3_C_2_30. The •OH generation of (D) HN-Ti_3_C_2_30 and (E) HNTM under US detecting by the fluorescence spectra of TA. (F) Adsorption of NBT treated by HN-Ti_3_C_2_30 for 6 min under US. (G) Spread plate and (H) the number of MRSA colonies of Control, US, Ti_3_C_2_ + US, HNTM + US, HN-Ti_3_C_2_10 + US, HN-Ti_3_C_2_30 + US, HN-Ti_3_C_2_50 + US. (I) Antibacterial mechanism. n = 3 independent experiments per group, ****P < 0.0001.

We examined the ROS (^1^O_2_, •O_2_-, and •OH) generation capacity of the individual samples under US irradiation (1.5 W/cm^2^, 50% duty cycle, 1 MHz) using various reagents. The production of ^1^O_2_ can be detected using DMA because it reacts with ^1^O_2_ and reduces the fluorescence intensity of DMA. The decrease in fluorescence in the HN-Ti_3_C_2_ group **(Figure 3B)** was more obvious than HNTM group **(Figure 3A)** within 6 min of US irradiation, indicating that the ^1^O_2_ production ability of HNTM was greatly enhanced after combination with Ti_3_C_2_. The curve of fluorescence intensity with time under US irradiation also supports the above results **(Figure 3C)**. To detect the generation of •OH, we used terephthalic acid (TA), which can be oxidized by •OH to generate 2-hydroxy-TA with a fluorescence peak at approximately 450 nm. As shown in **Figure 3D** and **E**, the fluorescence curve did not rise at 450 nm during 12 min of US irradiation, indicating that neither HNTM nor HN-Ti_3_C_2_ could produce •OH under US irradiation. Nitro blue tetrazolium (NBT) was used to detect ^•^O_2_^-^ because it reacts with ^•^O_2_^-^ to produce monoformazan (MF). The results showed that, under US irradiation, the ^•^O_2_^-^ generation ability of HNTM was weaker than that of HN-Ti_3_C_2_ (**Figure 3F** and **Figure S2C**). As for Ti_3_C_2_ NSs, almost no ROS production was detected under US irradiation (**Figure S2A, B** and **D**).

### *In vitro* antibacterial experiments

The excellent sonocatalytic activity and ROS generation ability of HN-Ti_3_C_2_ encouraged us to examinate the *in vitro* antibacterial efficiency of each sample against MRSA under US irradiation (1.5 W/cm^2^, 50% duty cycle, and 1 MHz) using a spreading plate experiment **(Figure 3G)**. Compared with HNTM group, the antibacterial efficiency was significantly improved in HN-Ti_3_C_2_ groups **(Figure 3H)**. And HN-Ti_3_C_2_30 has the highest antibacterial efficiency of up to 99.75%, which is higher than many other previous studies 53, 54. This indicates that HN-Ti_3_C_2_ has the potential to be used *in vivo* as an anti-MRSA therapy. Ti_3_C_2_ NSs greatly enhance the antimicrobial efficiency of HNTM because they can construct Schottky heterojunctions with HNTM and achieve rapid electron transfer, which greatly improves ROS production **(Figure 3I)**.

### *In vitro* cytocompatibility and osteogenesis

**Figure 4:**
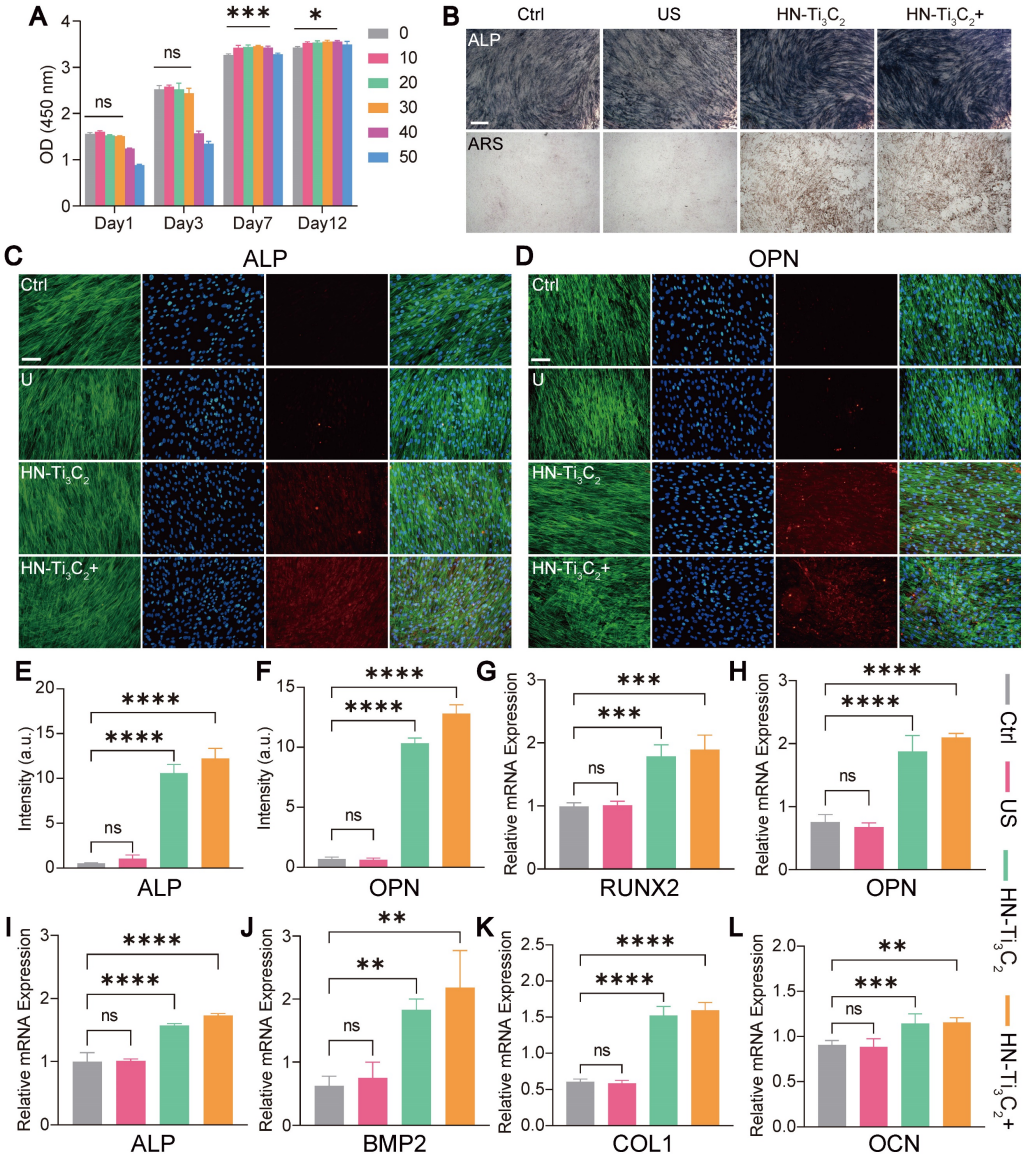
** Cell viability and *in vitro* osteogenesis performance.** (A) The cytocompatibility of HN-Ti_3_C_2_ detecting by CCK-8 assay. (B) ALP staining and ARS staining images of hBMSCs cultured in different conditions (Control, US, HN-Ti_3_C_2_ and HN-Ti_3_C_2_+US) after 21 days. Scar bar: 50 μm. (C, D) ALP and OPN immunofluorescence staining images of hBMSCs cultured in different conditions (Control, US, HN-Ti_3_C_2_ and HN-Ti_3_C_2_+US) after 21 days (green fluorescence: cytoskeleton; blue fluorescence: nucleus; red fluorescence: ALP or OPN). Scar bar: 50 μm. Quantitative analysis of (E) ALP and (F) OPN fluorescence intensity after 21 days. (G, H, I, J, K, L) RUNX2, OPN, ALP, BMP2, COL1 and OCN qRT-PCR results of hBMSCs treated in different conditions (Control, US, HN-Ti_3_C_2_ and HN-Ti_3_C_2_+US) after 14 days. n = 3 independent experiments per group, *P < 0.05, **P < 0.01, ***P < 0.001, ****P < 0.0001, ns = not significant.

CCK-8 assay was used to verify the effect of HN-Ti_3_C_2_ on the viability of hBMSCs *in vitro* **(Figure 4A)**. Surprisingly, we found that HN-Ti_3_C_2_ with concentration of 10, 20, 30, 40 μg/mL significantly promoted cell proliferation after 7 and 12 days compared with control. To test the potential of HN-Ti_3_C_2_ in repairing bone defects, we examined the osteogenic ability of HN-Ti_3_C_2_ *in vitro*. hBMSCs were co-cultured with HN-Ti_3_C_2_ for 14 and 21 days under US treatment (0.2 W/cm^2^, 50% duty cycle, 1 MHz), followed by alkaline phosphatase (ALP) and Alizarin Red S (ARS) staining. For ALP, the staining was significantly more pronounced in the HN-Ti_3_C_2_+US group compared to that of the control and US groups. Similar results were observed for ARS staining. The mineral nodules were higher in the HN-Ti_3_C_2_ and HN-Ti_3_C_2_+US groups than in the control and US groups and was highest in the HN-Ti_3_C_2_+US group **(Figure 4B and Figure S3A)**. These results indicated that both the HN-Ti_3_C_2_ and HN-Ti_3_C_2_+US treatments could effectively promote the osteogenic differentiation, and the HN-Ti_3_C_2_+US treatment was more obvious. This may be due to the up-regulation of intracellular Ca^2+^ accumulation by sonocurrent-induced charge transfer, which activates the calcium-induced osteogenic signaling pathway, thereby enhancing the osteogenic ability of HN-Ti_3_C_2_ under US irradiation 55-58. To verify the above conclusion, we conducted immunofluorescence staining to observe the expression of ALP protein **(Figure 4C and Figure S4A)** and osteopontin (OPN) (**Figure 4D and Figure S4C**). The results showed that after 14 and 21 days, the protein expression levels of ALP and OPN in the US group were not significantly different compared with control. However, the expression of ALP and OPN significantly increased after co-culture with HN-Ti_3_C_2_, and the increase was more obvious in the HN-Ti_3_C_2_+US group. The fluorescence quantitative statistical analysis also confirmed the above results **(Figure 4E, F and Figure S4B, D)**. Western blot was further carried out to detect the expression levels of osteogenesis-related proteins, including ALP, OPN, and RUNX2. As shown in **Figure S3B**, HN-Ti_3_C_2_ with or without US treatment, significantly increased the expression of the above proteins after 14 days. These results indicate that HN-Ti_3_C_2_ has a superior ability to promote osteogenic differentiation of hBMSCs under US treatment. To validate the ability of HN-Ti_3_C_2_ to promote osteogenic differentiation at the gene level, qPCR was performed to detect the expression levels of osteogenesis-related genes, including RUNX2, OPN, ALP, BMP2, COL1, and OCN. The results showed that HN-Ti_3_C_2_, with or without US treatment, significantly increased the expression levels of the above-mentioned osteogenesis-related genes after 7 and 14 days. There was no statistically significant difference between the US and control **(Figure 4G-L and Figure S5)**. The above results demonstrate the powerful ability of HN-Ti_3_C_2_ to promote osteogenic differentiation of hBMSCs under US irradiation, indicating its potential to accelerate bone defect repair.

### Osteogenesis mechanism

**Figure 5:**
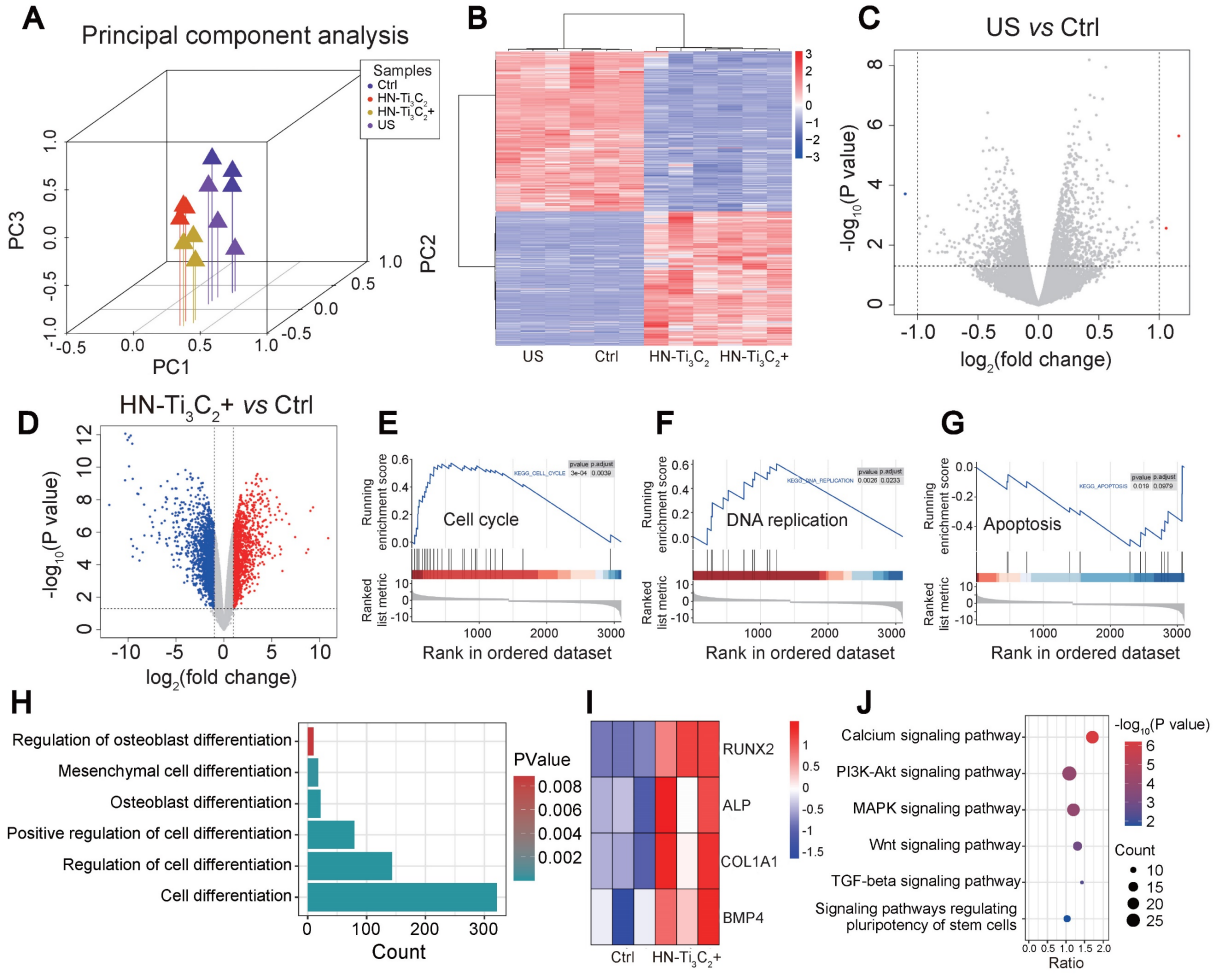
** Exploration of osteogenesis mechanism.** (A) Principal component analysis between different groups (Control, US, HN-Ti_3_C_2_ and HN-Ti_3_C_2_+US). (B) Heatmap showing differentially expressed genes (DEGs) between each group. (C) Volcano plot of US *vs* control. (D) Volcano plot of HN-Ti_3_C_2_+US *vs* control. (E, F, G) GSEA analysis of differentially expressed genes (DEGs). Plots are relative to (E) Cell cycle, (F) DNA replication and (G) Apoptosis. (H) GO pathways associated with cell differentiation. (I) DEGs associated with cell differentiation of HN-Ti_3_C_2_+US *vs* control. (J) KEGG enrichment analysis of HN-Ti_3_C_2_+US *vs* control. n = 3 independent experiments per group.

We used RNA sequencing to investigate the mechanism by which HN-Ti_3_C_2_ promoted the osteogenic differentiation of hBMSCs. Four groups (control, US, HN-Ti_3_C_2_, and HN-Ti_3_C_2_+US) were established for RNA sequencing. The principal component analysis showed good intragroup consistency in each group. As for the differences between groups, we found that the HN-Ti_3_C_2_ and HN-Ti_3_C_2_+US groups had significant differences compared to the control group **(Figure 5A)**. All differentially expressed genes (DEGs) among groups are shown in **Figure 5B**. There were large numbers of DEGs in the HN-Ti_3_C_2_ and HN-Ti_3_C_2_+US groups compared with control, while the gene expression was similar between US and control group. The volcano map also showed that compared with the control group, only a few DEGs in the US group was statistically different **(Figure 5C)**, while the expression of a large number of genes in the HN-Ti_3_C_2_ and HN-Ti_3_C_2_+US groups was statistically different **(Figure 5D and Figure S6A)**. These results indicate that HN-Ti_3_C_2_ can cause significant changes in gene expression in hBMSCs with or without US irradiation. GSEA showed that the genes with increased expression in the HN-Ti_3_C_2_ group were mainly related to the cell cycle **(Figure 5E)** and DNA replication **(Figure 5F)**, whereas the expression of genes related to apoptosis was downregulated **(Figure 5G)**. Heat maps revealed the significant upregulation of a large number DEGs involved in the cell cycle **(Figure S6B)**. Gene ontology (GO) analysis showed that differentially expressed genes between HN-Ti_3_C_2_ and the control group participated in the response to stimulation, regulation of biological and cellular processes, and biological regulation **(Figure S6C)**. KEGG pathway analysis indicated that numerous differentially expressed genes participated in DNA replication, the cell cycle, and apoptosis **(Figure S6D)**. These results indicate that HN-Ti_3_C_2_ can significantly promote the proliferation of hBMSCs, which is consistent with the CCK-8 and GSEA results.

Next, we analyzed the GO terms and genes involved in differentiation. We identified several GO terms associated with cell differentiation under US irradiation **(Figure 5H)** and found that several genes associated with osteogenic differentiation, including RUNX2, ALP, COL1A1, and BMP4, were significantly upregulated **(Figure 5I)**. KEGG enrichment analysis was performed to explore the signaling pathways related to osteogenic differentiation. The results showed that HN-Ti_3_C_2_ activated the calcium, MAPK, and Wnt signaling pathways under US irradiation, which played important roles in bone regeneration.

### *In vivo* treatment of osteomyelitis

**Figure 6:**
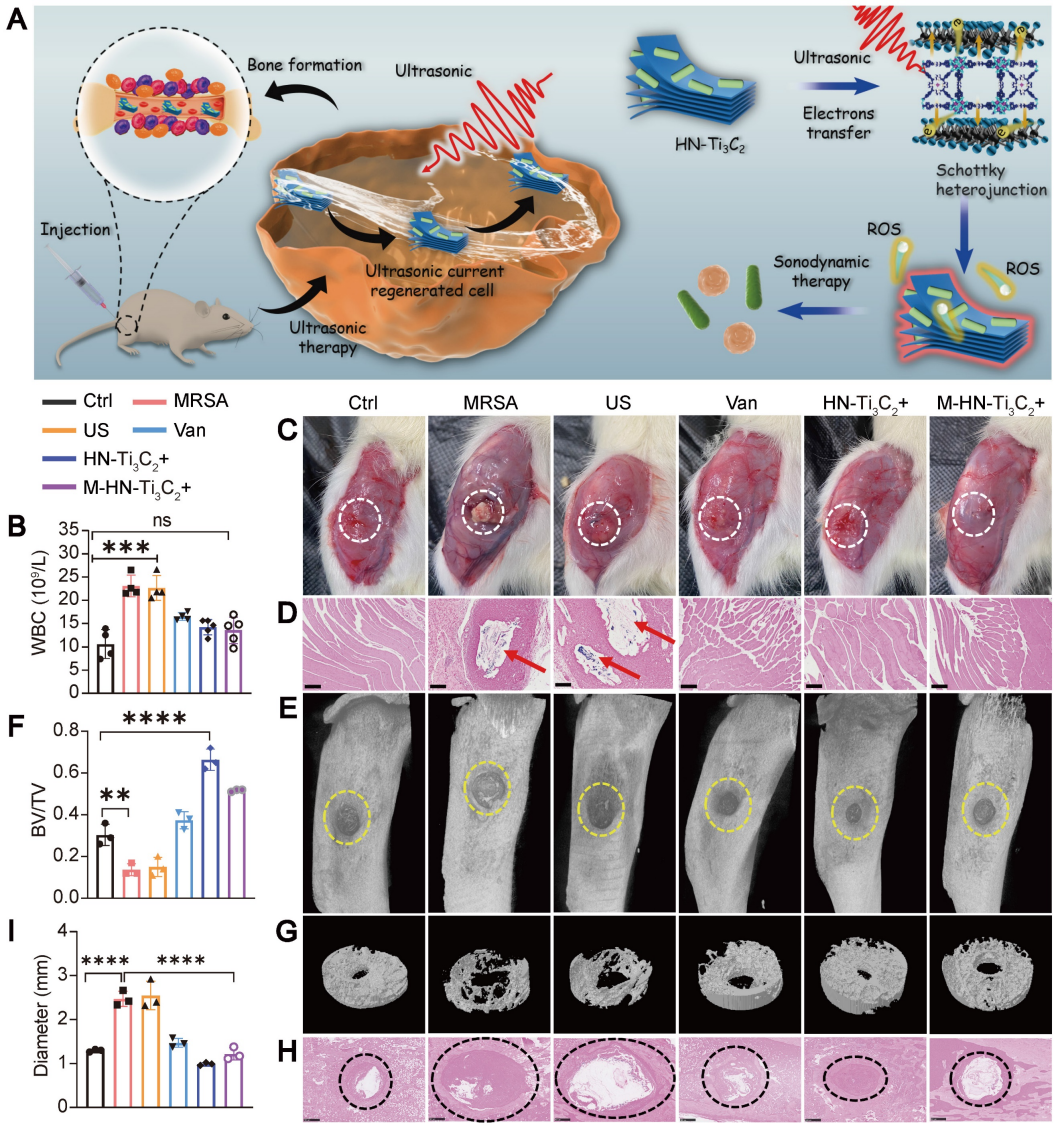
** Treatment of osteomyelitis.** (A) Schematic diagram of sonodynamic therapy for osteomyelitis using HN-Ti_3_C_2_ *in vivo*. (B) White blood cell count from blood routine examination. (C) Images of the surgical site of the infected leg. Abscesses are highlighted with white circles. (D) Gram stain images of muscle tissue near the sites of infection. MRSA are highlighted with red arrows. Scar bar: 500 μm. (E) Micro-CT analysis and (G) reconstruction results. The area of bone defect is circled with a yellow dotted-line. (F) Quantified BV/TV results of bone defects. (H) Gram stain images of infected bone. The sites of the bone defects were circled with a black dotted-line. Scar bar: 500 μm. (I) Quantitative statistics of bone defect size. n = 3 independent experiments per group, **P < 0.01, ***P < 0.001, ns = not significant.

We further explored the sonodynamic therapeutic effect of HN-Ti_3_C_2_ on osteomyelitis using a rat model of tibial osteomyelitis infection with MRSA **(Figure 6A)**. To evaluate the anti-infection ability of HN-Ti_3_C_2_ under US, we performed a white blood cell (WBC) test on postoperative day 28 to analyze the level of inflammation *in vivo* **(Figure 6B)**. The results showed that compared with the control, the WBC counts in the MRSA and US groups were significantly increased, whereas there was no statistical difference between the M-HN-Ti_3_C_2_+US group and the control group. We further studied the level of inflammation in infected bone tissue by Hematoxylin-eosin (HE) staining (**Figure S7A**). HE staining showed that a large number of inflammatory cells were observed in the MRSA group and the US group, while the inflammatory cell infiltration was significantly reduced in the M-HN-Ti_3_C_2_+US group. These results indicate that the treatment of HN-Ti_3_C_2_ with US reduces the level of inflammation *in vivo*, which is attributed to the strong antibacterial ability of HN-Ti_3_C_2_ under US irradiation. In addition, images of the muscles near the surgical site were obtained 28 days after surgery. As shown in **Figure 6C**, significant abscesses were observed in the MRSA and US groups but not in the remaining five groups. Gram-stained images of muscle tissue near the infection showed that large numbers of gram-positive bacteria-stained dark blue were observed only in the MRSA and US groups** (Figure 6D)**. These results indicated that HN-Ti_3_C_2_ effectively cleared MRSA infection under US irradiation. To confirm the ability of HN-Ti_3_C_2_ to promote bone defect repair, we performed micro-CT analysis of the bone defect sites. As shown in **Figure 6E and G**, owing to the bone destruction caused by MRSA infection, the bone defects in the MRSA and US groups were significantly enlarged compared with control group. The size of the bone defects in the vancomycin treatment (Van) group was similar to that in the control group. We found that HN-Ti_3_C_2_ reduced the area of bone defects compared to that of the control group, and the smallest bone defect area was observed in the HN-Ti_3_C_2_+US group without MRSA infection. This proves that HN-Ti_3_C_2_ has excellent bone repair ability in addition to its antibacterial ability under US irradiation. The results of the bone volume/total volume (BV/TV) analysis also showed the efficient bone repair ability of HN-Ti_3_C_2_, and it was more effective than many bone repair materials in other studies **(Figure 6F)** 59-61. Finally, we performed Gram staining on the infected bone defect sites and statistically analyzed the size of the bone defect. As shown in **Figure 6H and I**, the bone defects in the MRSA and US groups were larger than control group. In sharp contrast, HN-Ti_3_C_2_ significantly reduced the bone defect area. To study the osteogenic mechanism of HN-Ti_3_C_2_ *in vivo*, we performed immunohistochemical staining for ALP and OCN. As shown in **Figure S7B**, the expressions of ALP and OCN in HN-Ti_3_C_2_+US group and M-HN-Ti_3_C_2_+US group were significantly higher than those in other groups, indicating that HN-Ti_3_C_2_ has a strong ability to promote osteogenesis *in vivo* under US irradiation.

## Conclusion

In conclusion, we constructed an MXene-based Schottky heterojunction that concurrently augments anti-infection and bone regeneration properties. Ti_3_C_2_ improves the acoustic catalytic performance by rapidly transferring the charge carriers generated by HNTM under US irradiation and inhibiting the recombination of electron-hole pairs, which leads to the generation of large amounts of ROS to kill drug-resistant bacteria. HN-Ti_3_C_2_ generates a sonocurrent under low-intensity US that can promote bone regeneration. From the CCK-8 and RNA-seq results, we can see that HN-Ti_3_C_2_ may promote the proliferation of hBMSCs by regulating the cell cycle, DNA replication, and apoptosis. Moreover, after US irradiation, HN-Ti_3_C_2_ significantly accelerated the osteogenesis. KEGG analysis showed that HN-Ti_3_C_2_ promotes osteogenic differentiation by some key signaling pathways, including the calcium, Wnt, and TGF-beta signaling pathways. The above results indicate that HN-Ti_3_C_2_ can promote cell proliferation, and after low-intensity US irradiation, HN-Ti_3_C_2_ can promote bone regeneration through sonocurrent. This study provides an engineered HN-Ti_3_C_2_ nanocomposite for successful anti-infection treatment and bone regeneration. Unfortunately, because HN-Ti_3_C_2_ lacks the ability to target infectious lesions, it can only be used *in situ* to treat osteomyelitis, which will be the focus of our future research.

## Materials and Methods

### Preparation of HNTM

HNTM powder was prepared by heating ZrCl_4_ (Ourchem, 10026-11-6), benzoic acid (SCR, 65-85-0), and tetrakis (4-carboxyphenyl) porphyrin (TCPP, Aladdin, 14609-54-2) dissolved in DMF at 120 °C for 1 day. Briefly, ZrCl_4_ (10 mg) and benzoic acid (250 mg) were evenly dispersed in 2 mL of DMF and 200 μL of H_2_O under the action of an ultrasonic cleaner. TCPP powder (10 mg) was then added to the mixture and dispersed evenly under the action of an ultrasonic cleaner. The mixture was heated at 120 ℃ for 24 hours to obtain the HNTM solution. To obtain purple HNTM powder, the solution was first centrifuged to obtain the precipitate. The precipitate was cleaned and dried in a vacuum drying oven at room temperature.

### Preparation of HNTM-Ti_3_C_2_

HNTM and Ti_3_C_2_ powders were mixed in a specific mass ratio, evenly dispersed in ethanol using an ultrasonic homogenizer, and stirred overnight. The mixed solutions with different mass ratios were centrifuged at 12000 rpm for 5 min to obtain a precipitate, and HNTM-Ti_3_C_2_ powder with different mass ratios was obtained by drying in a vacuum drying oven at room temperature.

### Characterization of HNTM, Ti_3_C_2_, and HNTM-Ti_3_C_2_

The morphologies were characterized using HRTEM (JEM-2100F). A Zetasizer Nano (Malvern Zetasizer Nano ZS90, UK) was used to evaluate the zeta potentials. The crystal structures were analyzed by X-ray diffraction (XRD) (Bruker, D8A25, Germany). XPS spectra of the samples were obtained using an X-ray photoelectron spectrometer (Thermo Scientific, ESCALAB 250Xi, USA). Ultraviolet-visible diffuse reflectance spectra of the samples were recorded using a UV-vis-NIR spectrometer (Shimadzu, UV-3600, Japan). The PL spectra were recorded using a spectrofluorometer (Edinburgh, FLS1000, UK) under excitation at 425 nm.

### Detection of ROS production

The singlet oxygen production of samples under ultrasonic irradiation was detected by DMF solution of 9,10-dimethylanthracene (DMA). In short, the sample aqueous solution (200 μL, 1 mg/mL) was mixed with the DMA solution (200 μL, 200 μg/mL) and treated under US irradiation (1 MHz, 1.5 W/cm^-2^, 50% duty cycle). The decrease in DMA fluorescence intensity (at 0, 1, 2, and 6 min) was recorded to characterize ^1^O_2_ production. A DMSO solution of nitroblue tetrazolium (NBT) (2 μg/mL) was used to detect ^•^O_2_^-^ production. As NBT react with ^•^O_2_^-^ to generate MF, the production of ^•^O_2_^-^ can be detected by examining the absorption spectra of MF. Finally, an NaOH solution of terephthalic acid (TA) (600 μg/mL) was used as a probe to detect •OH production.

### Ultrasonic electrochemical measurements

The ultrasonic current and electrochemical impedance of the samples were measured in a Na_2_SO_4_ solution (0.1 M) using an electrochemical analysis instrument (CHI660E, China) under US (1 MHz, 1.5 W/cm^2^, continuous). An ethanol solution of the sample (100 μL, 2 mg/mL) was dropped onto a conductive glass and dried at room temperature for electrochemical measurements.

### *In vitro* antibacterial test

The spread plate method and MRSA suspensions (10^8^ CFU/mL) were used to study the antibacterial activities of the samples. Briefly, 10 μL of MRSA suspension was diluted 200-fold with each sample solution (500 μg/mL) and then treated for 15 min under US irradiation (1.0 MHz, 1.5 W/cm^2^, 50% duty cycle). Then, 20 μL suspensions were collected for the spread-plate experiment. The plates were then incubated at 37 °C in an incubator for 24 hours. The experiment was conducted using the following groups: control, US, and materials (HNTM, Ti_3_C_2_, HN-Ti_3_C_2_10, HN-Ti_3_C_2_30, HN-Ti_3_C_2_50) +US. Antibacterial efficiency was calculated following formula:

Antibacterial ratio (%) = (A_Ctrl_ - A_Material_)/A_Ctrl_ × 100%

### Isolation and culture of hBMSCs

Human bone marrow mesenchymal stem cells (hBMSCs) were extracted from the bone marrow blood of patients undergoing hip surgery at the Department of Orthopedics, Wuhan Union Hospital. All donors signed informed consent forms, and all procedures were in accordance with the Ethics Committee protocol of Tongji Medical College, Huazhong University of Science and Technology. Bone marrow blood was mixed with sterile PBS at an equal volume and added to human lymphocyte isolation medium (TBD Sciences, LTS1077) drop by drop at a volume ratio of 1:1. Then, hBMSCs were isolated by centrifugation (2500 rpm, 20 min). The hBMSC layer was then separated and added to a new centrifuge tube. hBMSCs were purified by centrifugation (1200 rpm, 5 min) and washed with sterile PBS. Finally, the cells were resuspended in DMEF/F12 medium (Gibco, C11330500BT) containing 10% fetal bovine serum (FBS, ScienCell, 0500), transferred into T25 culture flasks, and cultured at 37 ℃ in a 5% carbon dioxide cell incubator.

### CCK-8 assay

Cytocompatibility of the materials *in vitro* was verified using a CCK-8 kit. hBMSCs (15000 cells per well) were seeded in 24-well plates and co-cultured with different concentrations of materials (three repetitions were set for each concentration). CCK-8 assays were conducted after 1, 3, 7 and 12 days of culture. The specific experimental procedure was as follows: cell medium containing 10% CCK-8 solution (HYCEZMBIO, HYCCK8) was prepared, the medium containing materials was removed from the well plate and washed with PBS, and 400 μL of 10% CCK-8 medium was added to each well and incubated for 2.5 h at 37 °C. The liquid in the 24-well plate was transferred to a 96-well plate, and the absorbance of each well was measured at 450 nm by a VICTOR Nivo^®^ Multimode Plate Reader (PerkinElmer, Waltham, Massachusetts, USA).

### ALP staining

The ALP staining experiment groups were as follows: control, US, HN-Ti_3_C_2_, and HN-Ti_3_C_2_+US. ALP staining was performed after 14 and 21 days of co-culture of hBMSCs (1.5×10^4^ cells per well) seeded in 24-well plates with or without the material (30 μg/mL). During the incubation period, the US and HN-Ti_3_C_2_+US groups were treated with US every three days (four times, 1.0 MHz, 0.2 W/cm^2^, 50% duty cycle). The thickness of the medical ultrasonic coupler was 4 cm in all cell experiments, that is, the distance of the cell from the ultrasound source was 4 cm. Before staining with an ALP staining kit (Beyotime, C3206), the cells were washed thrice with PBS and fixed for 30 min in an appropriate amount of 4% paraformaldehyde.

### Alizarin Red S (ARS) staining

The grouping for the ARS was the same as that of ALP staining, and the cells were co-cultured and treated with US in the same manner. After 14 and 21 days of culture, staining was performed using a 0.2% Alizarin Red S solution (Solarbio, G1450). The cells were washed three times with PBS and fixed in 4% paraformaldehyde solution for 30 min before staining.

### Quantitative real-time polymerase chain reaction (qRT-PCR)

The following groups were assessed: control, US, HN-Ti_3_C_2_, and HN-Ti_3_C_2_+US. Cells seeded in 6-well plates were incubated for 7 or 14 days under the corresponding conditions according to the groups. The US and HN-Ti_3_C_2_+US groups were treated with US (1.0 MHz, 0.2 W/cm^2^, 50% duty cycle) every two days for 10 min each time, for a total of three times. After the cells were treated and cultured as described above, the cells were washed 3 times with PBS, the total RNA from each group was extracted using RNA-EasyTM Isolation Reagent (Vazyme, R701-01), and then the extracted RNA was purified with isopropanol and washed twice with 75% ethanol (prepared with ethanol and DEPC water). RNA was then dissolved in 20-50 μL of DEPC water (Biosharp, BL510A), and the RNA concentration was determined using an ultra-micro spectrophotometer. A 20 μL mixture of RNA solution, HiScript III RT SuperMix (Vazyme, R323-01), and DEPC water was prepared according to the measured RNA concentration and then reverse transcribed into complementary DNA (cDNA) using a SimpliAmp Thermal Cycler (Applied Biosystems, Massachusetts, USA). The prepared cDNA, primers, and SYBR fluorescent dye (Vazyme, Q712-02) were mixed in a certain proportion and transferred to enzyme-free 96-well plates, and real-time fluorescence changes were recorded using a CFX ConnectTM real-time System (BioRad Laboratories, California, USA). The recorded Cq values were normalized to GAPDH, and the gene expression of each group was calculated using the 2^-ΔΔ^Ct method.

### Immunofluorescent staining

This experiment was grouped into control, US, HN-Ti_3_C_2_, and HN-Ti_3_C_2_+US groups. hBMSCs seeded on glass coverslips in 24-well plates were incubated for 14 or 21 days under the corresponding conditions according to the groups. The US and HN-Ti_3_C_2_+US groups were treated with US (1.0 MHz, 0.2 W/cm^2^, 50% duty cycle) every two days for 10 min each time, for a total of 4 times. Cells were washed and fixed with 4% paraformaldehyde for 30 min before permeabilized with 0.5%Triton x-100 for 15 min. After sealing with blocking solution for 30 min, alkaline phosphatase (ALP; rabbit source, DF6225) and OPN (Affinity, rabbit source, AF0227) antibodies diluted in the antibody diluent were added to the corresponding wells and incubated for 12 h at 4 °C. After discarding the antibody, the wells were washed three times with 0.1% PBST and then anti-rabbit antibody (red fluorescence) (Proteintech, SA00013-4) was added to each well and incubated in the dark for 1 h at room temperature. Anti-rabbit antibody was discarded, and the wells were washed again with 0.1% PBST before staining the cytoskeleton and nucleus. The cytoskeleton was stained with green fluorescence-labeled phalloidin solution (Yeasen, 40735ES75), and the nuclei were stained with DAPI solution (Beyotime, P0131). Fluorescence staining images were captured using a fluorescence microscope.

### RNA sequencing

This experiment was grouped into control, US, HN-Ti_3_C_2_, and HN-Ti_3_C_2_+US groups. TRIzol reagent was used to extract total RNA from the above groups of hBMSCs, and the sample quality and integrity were tested. After quantitative analysis of the RNA samples, an RNA sequencing library was prepared. The Seqhalth Collaboration (Wuhan, China) assisted in processing the raw data, and we then used STRA software to map the human reference genome. The cutoff value for P value and old change were set as 0.05 and 2, respectively, and the DEGs were analyzed using the edgeR software package. The DEGs were then analyzed by GO and Genomes (KEGG) enrichment analyses using KOBAS software (version 2.1.1), and the p-value was set to 0.05.

### Animal experiment

We used a rat osteomyelitis model to verify the antibacterial and bone-repair abilities of the samples in each group *in vivo*. Male Sprague-Dawley (SD) rats weighing approximately 220 g were purchased from the Laboratory Animal Center of Huazhong Agricultural University, Wuhan. The animal experiments were approved by the Animal Research Committee of Tongji Medical College, Huazhong University of Science and Technology, Wuhan, China (No. S2807). Rats were randomly divided into six groups: control (Ctrl), MRSA, MRSA+US (US), MRSA+vancomycin (Van), HN-Ti_3_C_2_+US (HN-Ti_3_C_2_+), and MRSA+HN-Ti_3_C_2_+US (M-HN-Ti_3_C_2_ +). Rats were anesthetized and the rat osteomyelitis model was constructed as follows: First, the tibial plateau of the right leg of the rat was exposed and a 2 mm diameter bone defect was created using an electric drill. Then, the MRSA solution (100 μL, 10^8^ CFU/mL) was injected into the bone marrow cavity through the defect using a syringe. Finally, the bone defect was closed with bone wax to prevent the outflow of the MRSA solution. After establishing the model, different treatments were administered according to the above groups. The US parameters were set to 1.5 W/cm^2^, 50% duty cycle, 1 MHz, and irradiation for 15 min. The thickness of the medical ultrasonic coupler was 0.5 cm, that is, the distance between the skin and the ultrasound source was 0.5 cm. Vancomycin solution was administered to rats in the Van group at a dose of 40 mg/kg via the tail vein, whereas HN-Ti_3_C_2_ solution (500 μg/mL, 300 μL) was injected into the bone marrow cavity together with MRSA solution.

### Micro-CT

The infected tibia was scanned using a micro-CT system (Bruker, Skyscan1176), 3D digital images were constructed using 3D reconstruction software, and BV/TV values were obtained using CT analysis software.

### Statistical analysis

GraphPad Prism 9 and Origin 2021 software were used to analyze the data and presented as the mean ± standard deviation. All experiments were performed more than three times. Normality tests were performed in all experiments. We performed significance analyses by Student's *t*-test, one-way analysis of variance (ANOVA), and two-way analysis of variance. *P < 0.05, **P < 0.01, ***P < 0.001 and ****P < 0.0001 were considered statistically significant.

## Supplementary Material

Supplementary figures.

## Abbreviations

ALP: alkaline phosphatase
ANOVA: one-way analysis of variance
ARS: Alizarin Red S
BMP: bone morphogenetic protein
BV/TV: bone volume/total volume
CCK-8: cell Counting Kit-8
cDNA: complementary DNA
COL: collagen
CT: computed tomography
DAPI: 4',6-diamidino-2-phenylindole
DEGs: differentially expressed genes
DLS: dynamic light scattering
DMA: 9,10-dimethylanthracene
DMF: N,N-Dimethylformamide
FBS: fetal bovine serum
GAPDH: glyceraldehyde-3-phosphate dehydrogenase
GO: gene ontology
GSEA: gene set enrichment analysis
hBMSCs: human bone marrow mesenchymal stem cells
HE: hematoxylin-eosin
HNTM: zirconium-porphyrin-based metal-organic framework hollow nanotubes
KEGG: Kyoto Encyclopedia of Genes and Genomes
MAPK: mitogen-activated protein kinase
MF: monoformazan
MOFs: metal-organic frameworks
MRSA: methicillin-resistant *Staphylococcus aureus*
NBT: Nitro blue tetrazolium
NIR: near infrared
NSs: nanosheets
OCN: osteocalcin
OPN: osteopontin
PBS: phosphate buffered saline
PCN: porous coordination network
PDT: photodynamic therapy
PL: photoluminescence
QDs: quantum dots
qRT-PCR: quantitative real-time polymerase chain reaction
RNA-seq: RNA sequencing
ROS: reactive oxygen species
RUNX2: Runt-related transcription factor 2
SDT: sonodynamic therapy
TA: terephthalic acid
TCPP: tetrakis (4-carboxyphenyl) porphyrin
TEM: transmission electron microscopy
TGF: transforming growth factor
Ti_3_C_2_: titanium carbide
US: ultrasound
UV-vis: ultraviolet-visible
WBC: white blood cell
XPS: X-ray photoelectron spectroscopy
XRD: X-ray diffraction
^1^O_2_: singlet oxygen
2D: two-dimensional
3D: three-dimensional
•O_2_-: superoxide anion
•OH: hydroxyl radical
