# Ultrasound-controlled MXene-based Schottky heterojunction improves anti-infection and osteogenesis properties: Erratum

**DOI:** 10.7150/thno.119205

**Published:** 2026-01-01

**Authors:** Hongchuan Wang, Na Mu, Yaqi He, Xiaoguang Zhang, Jie Lei, Cao Yang, Liang Ma, Yong Gao

**Affiliations:** 1 Department of Orthopaedics, Union Hospital, Tongji Medical College, Huazhong University of Science and Technology, Wuhan 430022, China.; 2 College of Agronomy, Xinjiang Agriculture University, Urumqi, Xinjiang, China.

The authors apologize that a incorrect representative image was accidentally used in our previously published paper when the first author assembled the figures. Specifically, in the OCN immunohistochemical staining results of Supplementary Figure S7B, the image of the US group was wrongly used. The corrected figure is shown below. The authors declare that this correction does not change the results or conclusions of their paper. The authors sincerely apologize to the Journal and its readers for the confusion this may have caused.

## Figures and Tables

**Figure A:**
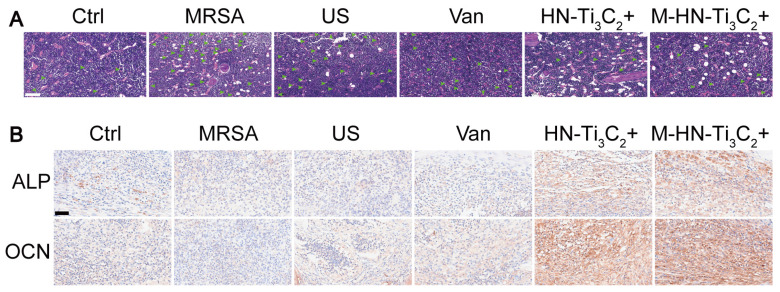
Corrected figure for original Figure S7.

