# Supplementary material for: Ultrasound-controlled MXene-based Schottky heterojunction improves anti-infection and osteogenesis properties
Source: Theranostics. 2023 Mar 13;13(5):1669–83. doi: 10.7150/thno.81511 (PMC10086208; doi:10.7150/thno.81511)
Supplement: Supplementary file 1 — Supplementary figures. [file thnov13p1669s1.pdf]

Supplementary Information

Ultrasound-controlled MXene-based Schottky heterojunction improves anti-infection and osteogenesis properties.

Hongchuan Wang [a,1], Na Mu [b,1], Yaqi He [a,1], Xiaoguang Zhang [a], Jie Lei [a], Cao Yang [a,], Liang Ma [a,*], Yong Gao [a,*]*

[a] *Department of Orthopaedics, Union Hospital, Tongji Medical College, Huazhong University of Science and Technology, Wuhan 430022, China.*

[b] *College of Agronomy, Xinjiang Agriculture University, Urumqi, Xinjiang, China.*

[1] The three authors share the co-first authorship due to their equal contribution.

* To whom correspondence should be addressed.

docgao@163.com (Y. Gao) ; D202181825@hust.edu.cn (L. Ma) ;

caoyangunion@hust.edu.cn (C. Yang)

Figure captions

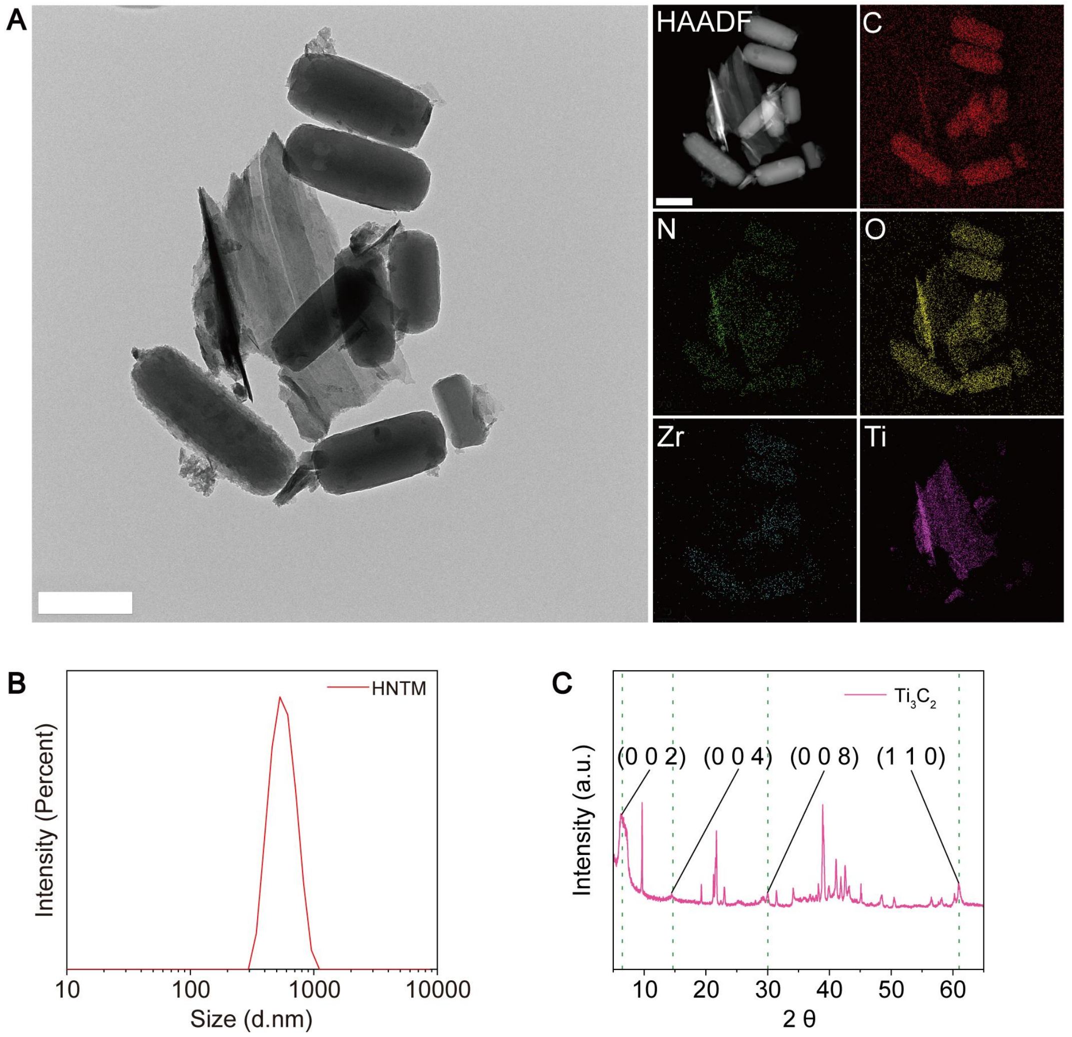

Figure S1. (A) TEM image and Energy-dispersive X-ray spectroscopy elemental mapping of HN-Ti_3C_2 after 7 days of immersion in PBS solution. Scar bar: 500 nm. (B) Dynamic light scattering (DLS) size distribution of HNTM. (C) XRD pattern of Ti_3C_2.

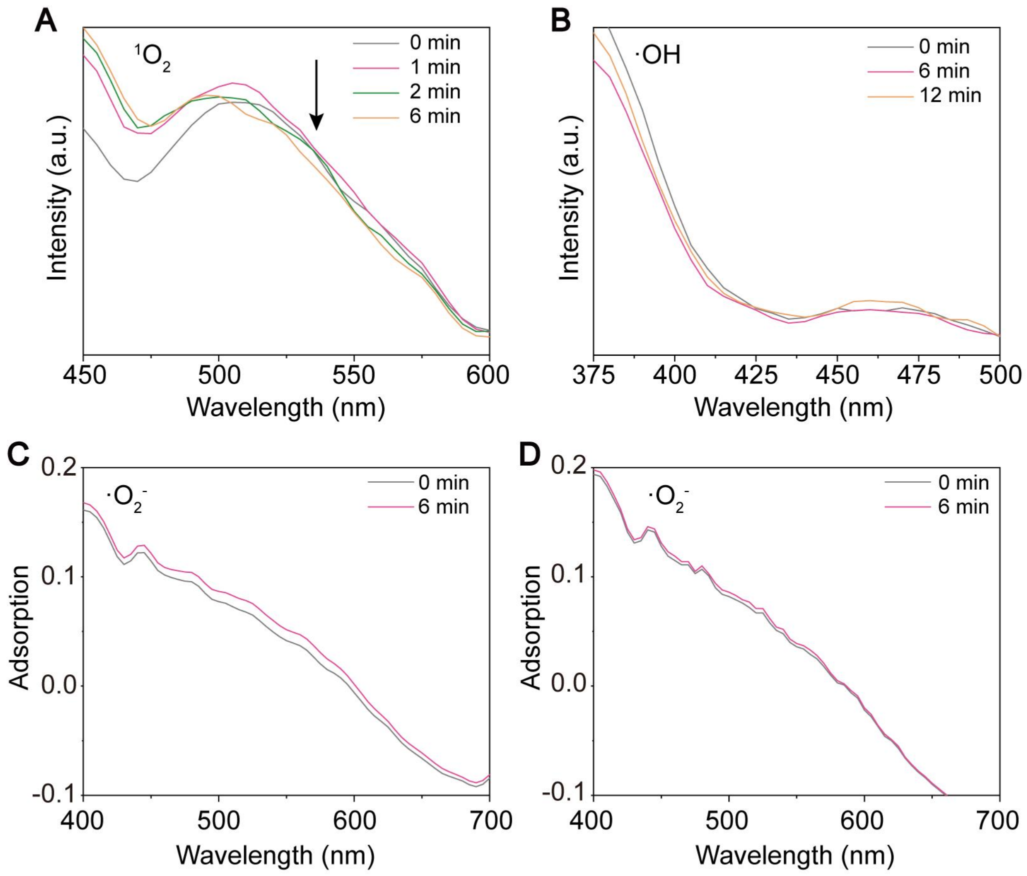

Figure S2. (A) The $^1O_2$ generation of Ti_3C_2 under US detecting by the decrease of DMA fluorescence intensity. (B) The •OH generation of Ti_3C_2 under US detecting by the fluorescence spectra of TA. Adsorption of NBT treated by (C) HNTM or (D) Ti_3C_2 for 6 min under US.

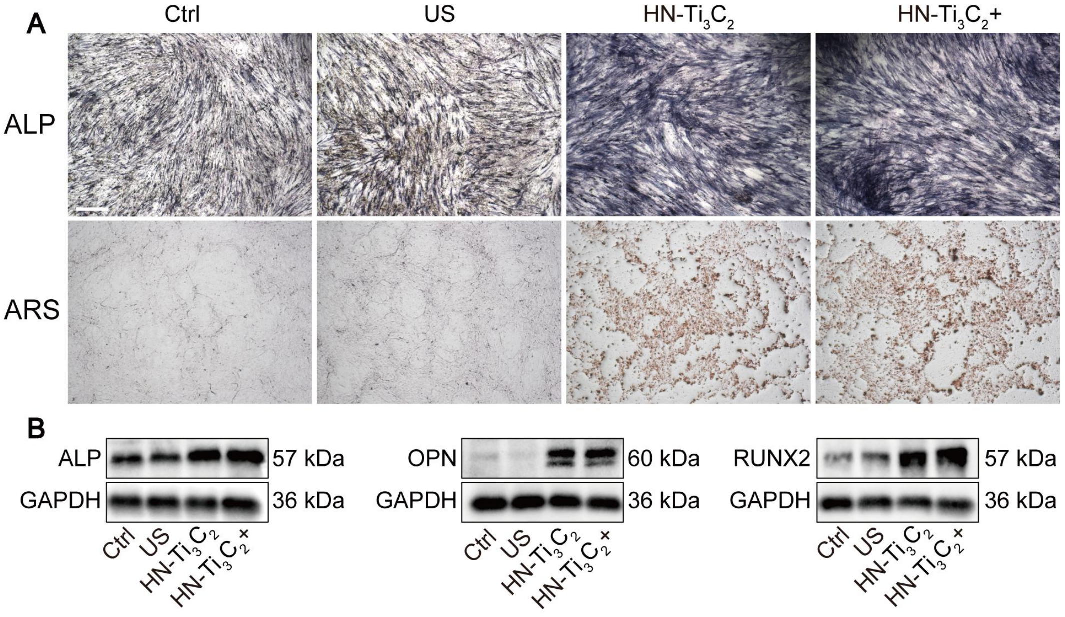

Figure S3. (A) ALP staining and ARS staining images of hBMSCs cultured in different conditions (Control, US, HN-Ti_3C_2 and HN-Ti_3C_2+US) after 14 days. Scar bar: 50 μm. (B) Western blot of ALP, OPN, and RUNX2 proteins of hBMSCs treated in different conditions (Control, US, HN-Ti_3C_2 and HN-Ti_3C_2+US) after 14 days.

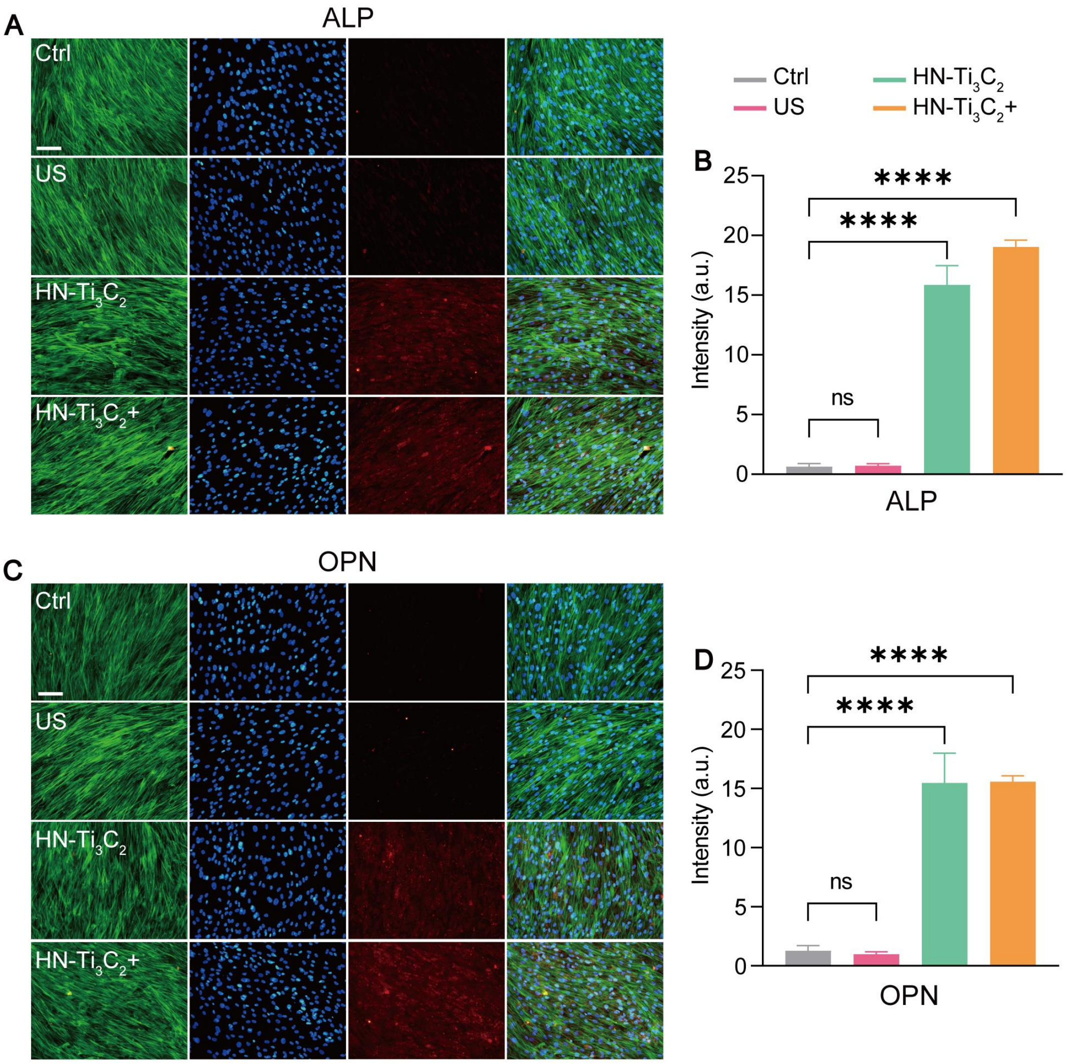

Figure S4. (A, C) ALP and OPN immunofluorescence staining images of hBMSCs cultured in different conditions (Control, US, HN-Ti_3C_2 and HN-Ti_3C_2+US) after 14 days (green fluorescence: cytoskeleton; blue fluorescence: nucleus; red fluorescence: ALP or OPN). Scar bar: 50 μm. Quantitative analysis of (B) ALP and (D) OPN fluorescence intensity after 14 days. n = 3 independent experiments per group, **P* < 0.05, ***P* < 0.01, ****P* < 0.001, *****P* < 0.0001, ns = not significant.

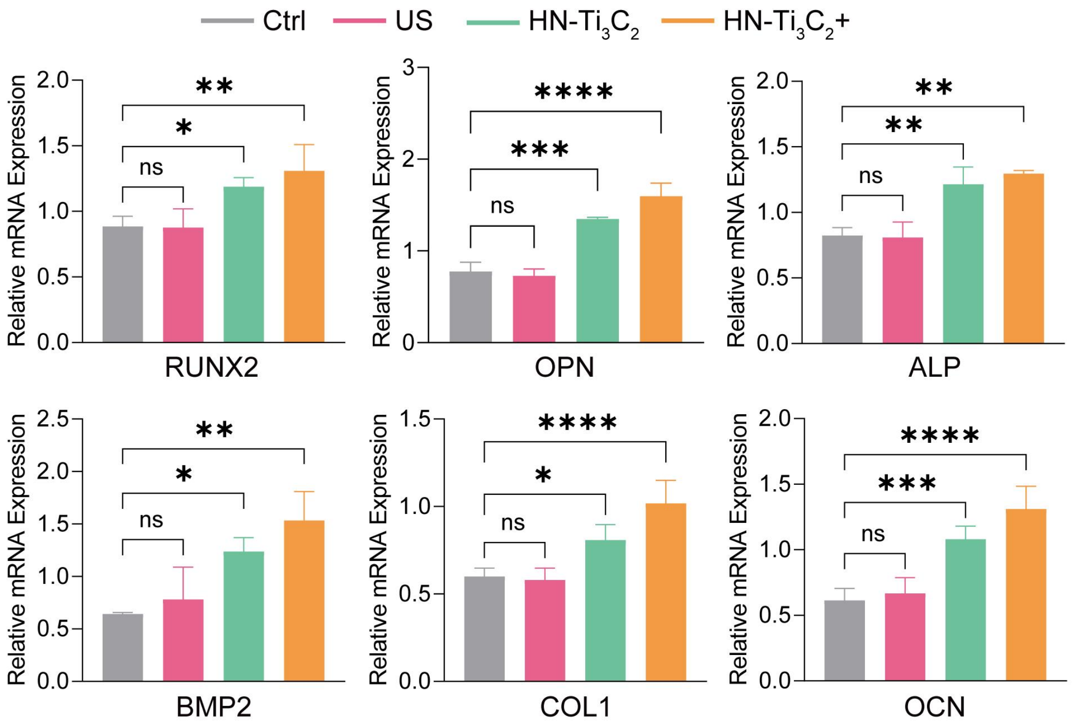

Figure S5. qRT-PCR results of RUNX2, OPN, ALP, BMP2, COL1 and OCN treated in different conditions (Control, US, HN-Ti_3C_2 and HN-Ti_3C_2+US) after 7 days. n = 3 independent experiments per group, *$P < 0.05$, **$P < 0.01$, ***$P < 0.001$, ****$P < 0.0001$, ns = not significant.

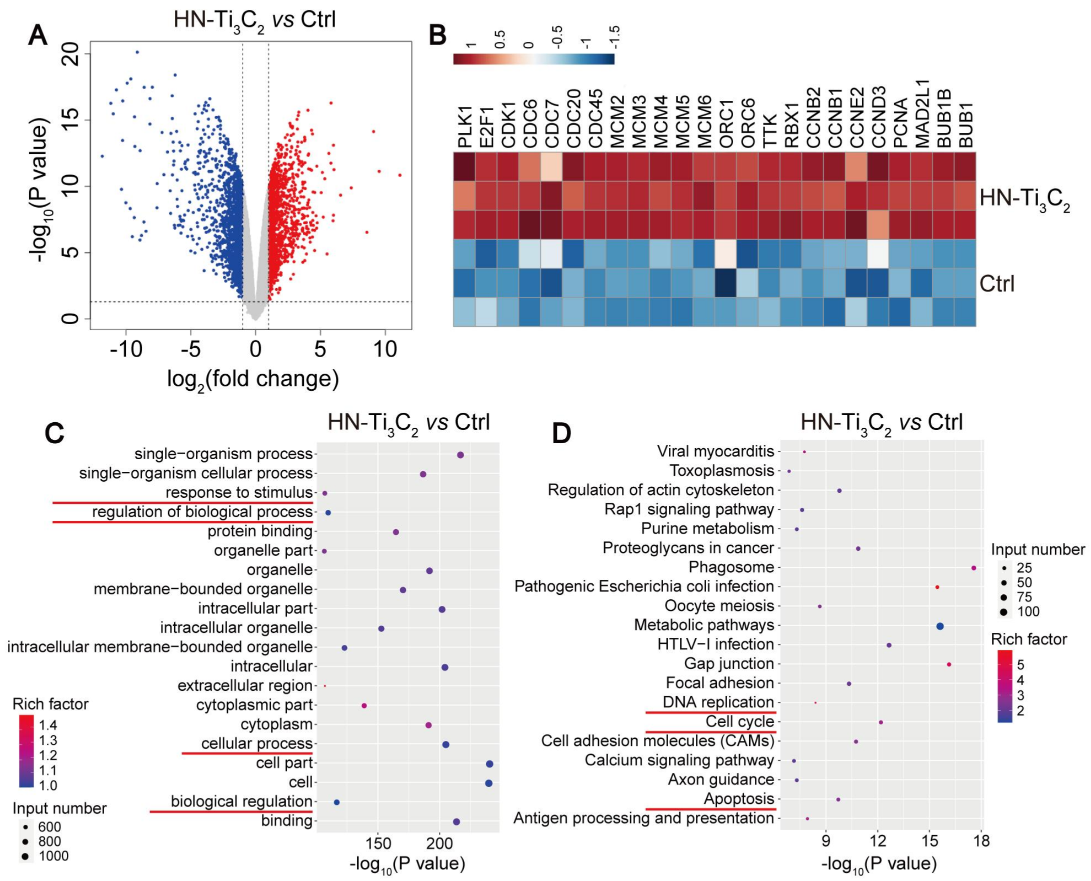

Figure S6. (A) Volcano plot of HN-Ti_3C_2 *vs* control. (B) Heatmap of cell cycle related genes. (C) The top 20 most significantly enriched GO pathways of HN-Ti_3C_2 *vs* control. (D) The top 20 most significantly enriched KEGG pathways of HN-Ti_3C_2 *vs* control. n = 3 independent experiments per group.

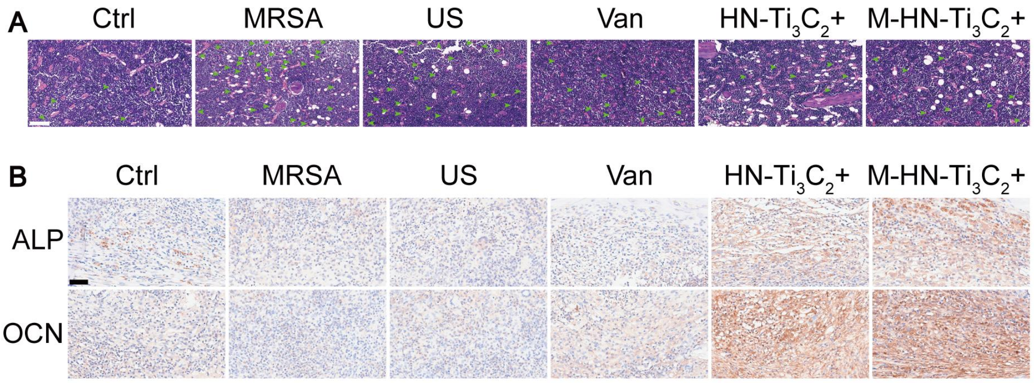

Figure S7. (A) HE staining of infected bone. Inflammatory cells are highlighted with green arrows. Scar bar: 100 μm. (B) Immunohistochemical staining of ALP and OCN. Scar bar: 50 μm.
